# MPSA-Conformer-CTC/Attention: A High-Accuracy, Low-Complexity End-to-End Approach for Tibetan Speech Recognition

**DOI:** 10.3390/s24216824

**Published:** 2024-10-24

**Authors:** Changlin Wu, Huihui Sun, Kaifeng Huang, Long Wu

**Affiliations:** 1 School of Mechanical and Electrical Engineering, Huainan Normal University, Huainan 232001, China; wuchanglin@hnnu.edu.cn (C.W.); hkf@hnnu.edu.cn (K.H.); lwu@hnnu.edu.cn (L.W.); 2 Human–Computer Collaborative Robot Joint Laboratory of Anhui Province, Hefei 230002, China

**Keywords:** Tibetan speech recognition, end-to-end models, conformer architecture, connectionist temporal classification (CTC)

## Abstract

This study addresses the challenges of low accuracy and high computational demands in Tibetan speech recognition by investigating the application of end-to-end networks. We propose a decoding strategy that integrates Connectionist Temporal Classification (CTC) and Attention mechanisms, capitalizing on the benefits of automatic alignment and attention weight extraction. The Conformer architecture is utilized as the encoder, leading to the development of the Conformer-CTC/Attention model. This model first extracts global features from the speech signal using the Conformer, followed by joint decoding of these features through CTC and Attention mechanisms. To mitigate convergence issues during training, particularly with longer input feature sequences, we introduce a Probabilistic Sparse Attention mechanism within the joint CTC/Attention framework. Additionally, we implement a maximum entropy optimization algorithm for CTC, effectively addressing challenges such as increased path counts, spike distributions, and local optima during training. We designate the proposed method as the MaxEnt-Optimized Probabilistic Sparse Attention Conformer-CTC/Attention Model (MPSA-Conformer-CTC/Attention). Experimental results indicate that our improved model achieves a word error rate reduction of 10.68% and 9.57% on self-constructed and open-source Tibetan datasets, respectively, compared to the baseline model. Furthermore, the enhanced model not only reduces memory consumption and training time but also improves generalization capability and accuracy.

## 1. Introduction

Speech recognition, as a core technology in human–computer interaction and intelligent speech processing, plays an increasingly important role. Since the development of the first Automatic Speech Recognition (ASR) system by Bell Labs in 1952 [1], speech recognition technology has undergone over 70 years of evolution. Significant advancements have been made in languages such as English, Mandarin Chinese, and German, particularly in areas such as speech database construction, recognition accuracy, and speech interaction applications [2]. In contrast, research on Tibetan speech recognition technology has only emerged in recent years. Tibetan exhibits numerous differences from mainstream languages like Chinese and English, including tonal variations, initial consonants, vowel endings, tonal markers, and digraphs. These factors contribute to the complexity and variability of Tibetan pronunciation, posing significant challenges for Tibetan speech recognition.

In 2014, Alex Graves [3] achieved end-to-end (E2E) speech recognition [4], leading to its widespread application in the field. The input to an E2E system consists of acoustic features or speech waveforms. Unlike traditional methods, E2E simplifies the integration of acoustic models, language models, and pronunciation dictionaries [5] into a sequence-to-sequence recognition module, enabling a direct conversion from speech to text. Currently, popular E2E speech recognition methods are primarily built upon three models: Connectionist Temporal Classification (CTC) [6,7], attention-based encoder–decoder (AED) [8,9], and Conformer [10,11]. These deep learning models are relatively easy to construct and fine-tune, and in certain application scenarios, they achieve recognition rates that surpass those of traditional speech recognition models. Furthermore, they enable flexible combinations of multiple models, leveraging the strengths of different foundational models to achieve improved performance [12,13].

In recent years, significant progress has been made in the field of Tibetan speech recognition, primarily utilizing techniques based on Connectionist Temporal Classification (CTC) and attention mechanisms. Wang Qingnan et al. developed a CTC-based continuous speech recognition system for Tibetan, which integrates linguistic knowledge of the Tibetan language to enhance the discriminative power and decoding speed of the acoustic model [14]. Following this, Zhao et al. proposed a high-accuracy recognition model for Lhasa Tibetan using an active learning approach for speech corpus selection, reducing manual annotation complexity [15]. In the same year, Huang et al. achieved end-to-end acoustic modeling for Tibetan speech using Recurrent Neural Networks (RNN) and continuous temporal classification algorithms [16]. In the improvements made to the CTC model, Zhao et al. introduced multi-task learning, resulting in a WaveNet-CTC model that demonstrated superior recognition performance [17]. Zhou et al. incorporated an attention mechanism into their model, achieving a word error rate (WER) of 38.64% [18]. Additionally, Nan et al. combined RNNs to propose the BLSTM-CTC and RNN-BLSTM-ReLU models [19], further enhancing recognition accuracy. Yue et al. employed dilated convolutions within the WaveNet-CTC architecture, achieving WERs of 28.83%, 62.56%, and 17.6% for the Lhasa, Kham, and Amdo dialects, respectively, [20], while Guo et al. proposed a CNN-BLSTM-CTC framework that attained a WER of 35.51% for the Lhasa dialect [21].

In attention-based models, Huang explored the application of convolutional networks in speech recognition and proposed a dropout-enhanced CTC-CNN network, achieving a word error rate (WER) of 15.4% for the Amdo dialect [22]. Sun introduced a hybrid CTC-attention model, which attained a WER of 31.5% for the Amdo dialect [23]. In the realm of multi-feature and multi-modal methods, Gao et al. developed the AV-WaveNet-CTC-A-I-10 model that combines audio and video features, achieving a WER of 42.7% for the Lhasa dialect [24]. Hou proposed a CNN-based multi-feature acoustic model, reaching a WER of 24.64% for the Lhasa dialect [25]. Suan Taiben utilized Tibetan characters, syllables, and Latin phonemes as modeling units, testing on a mixed dataset of the Wuzang and Amdo dialects, resulting in a WER of 19.26% [26]. Kang integrated pre-trained models with RNNs, proposing a Pre-Training+BiLSTM+Attention model that achieved a WER of 26.6% for the Amdo dialect [27]. Yang employed a Transformer model, which reached a WER of 25.8% for the Lhasa dialect [28]. Gong et al. used Latin phonemes, syllables, and Tibetan characters as modeling units, introducing a multi-scale feature fusion concept in the MRDCNN-CTC model, achieving a WER of 18.67% for the Amdo dialect [29]. Qin et al. improved end-to-end Tibetan speech recognition through multilingual and multi-layer unit modeling, resulting in a 14.2% reduction in character error rate (CER) compared to DNN-HMM models [30].

Despite the promising performance demonstrated by CTC-attention models in Tibetan speech recognition, several shortcomings remain. These models may encounter high computational complexity and inefficiencies in training when handling long sequence inputs, and their accuracy is often constrained by the assumption of conditional independence. Moreover, the conditional independence assumption of CTC can lead to a decline in recognition rates, particularly in the absence of an additional language model. To address these challenges, this study proposes an enhanced Conformer-CTC/Attention model aimed at improving the accuracy of end-to-end Tibetan speech recognition while reducing computational complexity. The specific contributions of this work are as follows:

- Hybrid CTC-Attention Decoder: We introduce a decoding strategy that integrates Connectionist Temporal Classification (CTC) with Attention mechanisms, effectively leveraging the advantages of automatic alignment and attention weight extraction. Utilizing a Conformer as the encoder, we construct the Conformer-CTC/Attention model. This model first extracts global features from the speech signal using the Conformer, then jointly decodes the speech features through CTC and Attention to produce text sequences, resulting in enhanced accuracy and efficiency in speech recognition.
- Probabilistic Sparse Attention: To address convergence challenges during the training of the Conformer-CTC/Attention model, particularly with long input feature sequences, we observe that the time and space complexity of attention calculations increases quadratically with sequence length. We propose a Probabilistic Sparse Attention mechanism and apply it to the joint CTC/Attention decoding framework. Experimental results demonstrate that this approach effectively reduces computational complexity and memory usage while simultaneously enhancing the model’s performance and stability.
- Maximum Entropy-based CTC Improvement Algorithm: We present a maximum entropy-based improvement algorithm for CTC to mitigate issues related to the increasing number of paths, spike distributions, and local optima during training. By optimizing the training process, we reduce the impact of erroneous feedback, thereby enhancing the model’s robustness and training efficiency.

## 2. Relevant Knowledge

### 2.1. Conformer Encoder

**Figure 1:**
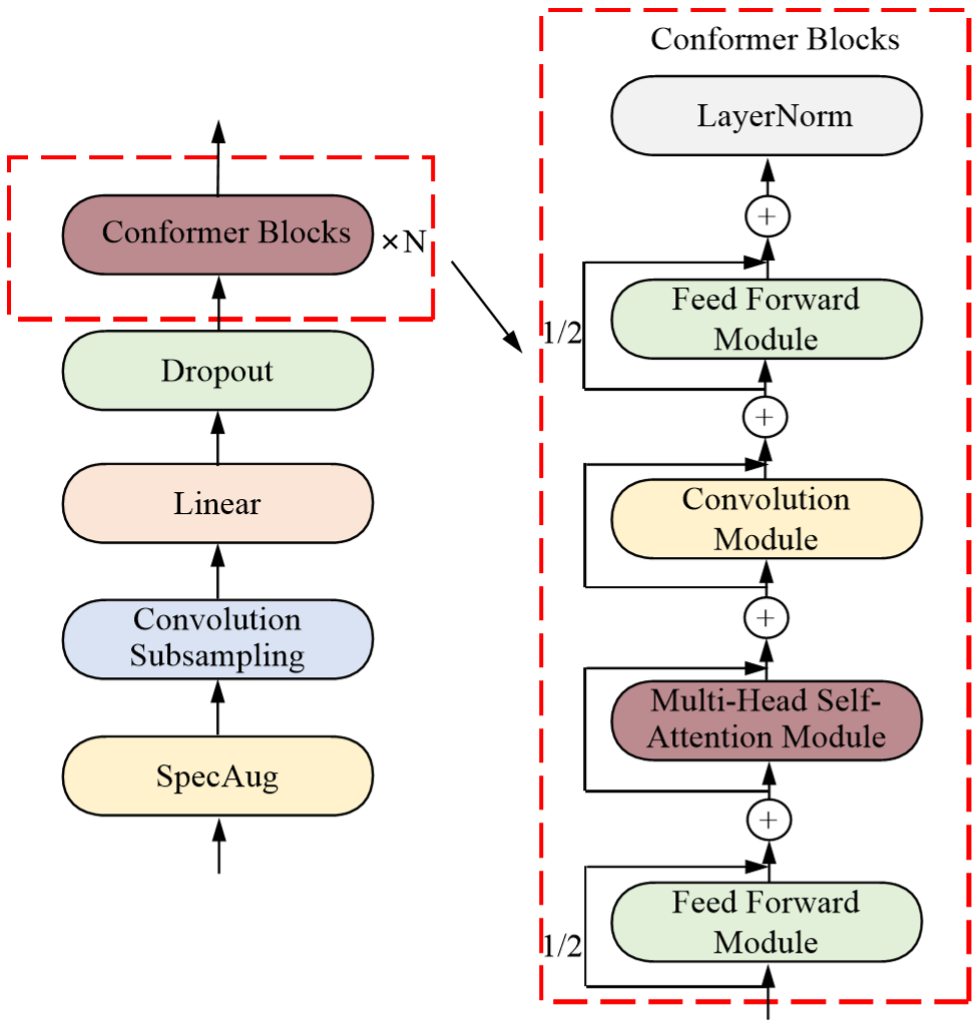
Structure of the Conformer Encoder Model.

The Conformer model proposed by Gulati et al. [31] combines convolution and self-attention, building on the work presented in the literature [12]. Self-attention captures global interactions, while convolution effectively captures local correlations based on relative shifts, resulting in more effective outcomes than using either convolution or self-attention alone. The acoustic input network structure of the Conformer encoder, as illustrated in Figure 1, consists of four main components: the SpecAugment module [32], a convolution module (Convolution Subsampling), a linear layer (Linear), and a Dropout layer. The SpecAugment module is responsible for data augmentation of the filter bank (FBank); the Convolution Subsampling performs downsampling; the Linear layer reduces the feature dimensions; and the Dropout layer helps mitigate overfitting, thus achieving a regularization effect. A series of Conformer Blocks are then employed to process the input. The left side of Figure 1, displays the overall architecture of the Conformer encoder, while the right side illustrates the specific structure of the Conformer Block.

The Conformer Block comprises three key components: a Feed Forward Module, a Multi-Head Self-Attention Module, and a Convolution Module. Each Conformer Block is flanked by a Feed Forward layer, with the Multi-Head Self-Attention and Convolution modules positioned centrally. The Feed Forward layers incorporate half-step residual connections, and Layer Normalization (LayerNorm) is applied following each major module. Additionally, residual connections are integrated within each module. This architectural design effectively combines convolution and attention mechanisms, leading to a substantial enhancement in overall performance.

The Multi-Head Self-Attention Module utilized in this study integrates a key technique from Transformer-XL [33]: the relative sinusoidal position encoding scheme. This relative position encoding enhances the generalization capability of the self-attention module across varying input lengths, resulting in an encoder that exhibits improved robustness to fluctuations in sequence length. The convolution module features a pointwise convolution with an expansion factor of 2, followed by a GLU activation layer that projects the channel dimensions. This is succeeded by a one-dimensional depthwise convolution, followed by Batch Normalization and a Swish activation layer. In the Conformer Block, identical Feed Forward Modules are positioned at both the beginning and the end, with each *FFN* contributing half of the output value, referred to as the half-step *FFN*. Mathematically, for the input $x_{i}$ of the *i*-th Conformer Block, the output $y_{i}$ is computed as follows:

(1) $${\tilde{x}}_{i}=x_{i}+\frac{1}{2}FFN\left(x_{i}\right)$$

(2) $$x\prime_{i}={\tilde{x}}_{i}+MHSA\left({\tilde{x}}_{i}\right)$$

(3) $$x{\prime \prime }_{i}=x\prime_{i}+Conv\left(x\prime_{i}\right)$$

(4) $$y_{i}=LayerNorm\left(x{\prime \prime }_{i}+\frac{1}{2}FFN\left(x{\prime \prime }_{i}\right)\right)$$

In this context, *FFN* refers to the value of the Feed Forward Module, *MHSA* denotes the Multi-Head Self-Attention Module, Conv indicates the Convolution Module, and LayerNorm represents Layer Normalization. Residual connections are employed between each module.

### 2.2. Attention Decoder

**Figure 2:**
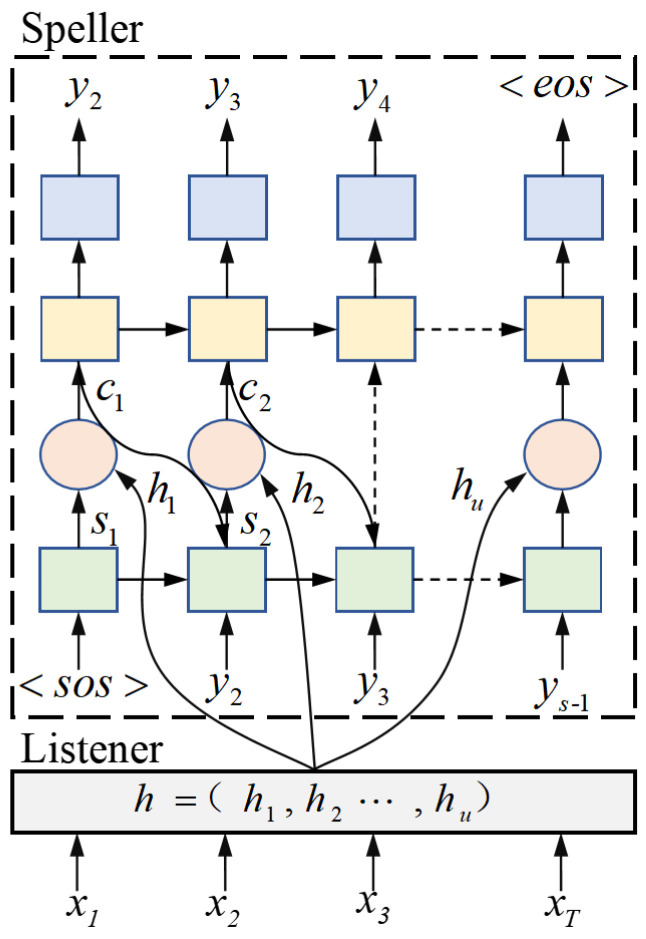
LAS model architecture.

The attention-based model was first introduced in speech recognition tasks by the Listen, Attend and Spell (LAS) model [34], proposed by William Chan et al. This model is a sequence-to-sequence architecture inspired by machine translation tasks. The LAS model comprises three components: ‘Listen’, which serves as the encoder based on a recurrent neural network (RNN), ‘Spell’, which functions as the decoder also based on an RNN, and ‘Attend’, which is the attention layer that establishes a mapping relationship between the encoder and the decoder, while the specific RNN architectures used for the encoder, decoder, and attention mechanism may vary across different papers, the overall framework remains consistent, as illustrated in Figure 2.

In Figure 2, the listener serves as the encoder of the acoustic model, performing the encoding operation that transforms the input acoustic sequence $x=(x_{1},x_{2},\ldots ,x_{T})$ into a high-level representation *h*. The length of the high-level feature sequence *h* can either match that of the input acoustic sequence *x* or produce a shorter sequence through downsampling.

The speller is a decoder based on the attention mechanism. At each output step, the transformer generates a probability distribution for the next character based on all previously observed characters, resulting in the probability of the output sequence *y* as follows:

(5) $$P\left(y|x\right)=P\left(y|h\right)=\prod_{t=1}^{T}P(y_{t}|h,y<t)$$

At each time step *t*, the attention mechanism computes the conditional dependence of the output on the encoder features *h*. The attention mechanism serves as a function of the current decoder hidden state and the encoder output features, compressing the encoder features into a context vector $c_{t}$ through the following process:

(6) $$u_{it}=v^{T}tanhW_{h}h_{i}+W_{d}d_{t}+b_{a}$$

In this context, the vectors $v^{T}$, $b_{a}$, and the matrices $W_{h}$ and $W_{d}$ are parameters learned during training; $d_{t}$ represents the hidden state of the decoder at time step *t*. The attention distribution is then obtained by applying the softmax function to $u_{it}$:

(7) $$a_{t}=softmax\left(u_{it}\right)$$

The context vector is obtained by weighting and summing the encoder features $a_{t}$ using the attention scores $h_{i}$:

(8) $$c_{t}=\sum_{i=1}^{K}a_{it}h_{i}$$

At each time step, the decoder’s hidden state $d_{t}$, which captures the context of previous outputs, is obtained as follows:

(9) $$d_{t}=RNN({\bar{y}}_{t-1},d_{t-1},c_{t-1})$$

In this context, $d_{t-1}$ represents the previous hidden state, and ${\bar{y}}_{t-1}$ is the embedding vector obtained from $y_{t-1}$. At time step *t*, the posterior probability of the output $y_{t}$ is expressed as follows:

(10) $$P\left(y_{t}|h,y<t\right)=softmax\left(W_{s}\left[c_{t};d_{t}\right]+b_{s}\right)$$

In this context, $W_{s}$ and $b_{s}$ are learnable parameters. Finally, the model’s loss function is defined as follows:

(11) $$L_{att}=-\ln\left(P\left(y|x\right)\right)$$

Although the LAS model, with its self-attention encoder–decoder structure, has achieved significant advancements in speech recognition tasks, it utilizes RNN network structures for encoding acoustic features and text features separately. This reliance on RNNs makes it challenging for the model to converge during training.

### 2.3. Connectionist Temporal Classification

The CTC (Connectionist Temporal Classification) model is a technique designed for end-to-end speech recognition, addressing the issue of mismatched lengths between input and output sequences [35]. Essentially, CTC serves as a loss function that is probabilistic in nature, aiming to establish a reliable correspondence between the input and output sequences.

In the CTC model, given an input sequence $X=[x_{1},x_{2},\ldots ,x_{T}]$ of length *T*, each time step *t* corresponds to an observed label. The actual output sequence is a sequence $Z=\left[z_{1},z_{2},\ldots ,z_{r}\right]$ of length *r*, where each time step *t* corresponds to either a character label or a blank character. The model’s predicted sequence $C=[c_{1},c_{2},\ldots ,c_{T}]$ is also of length *T*, where $c^{t}$ represents a probability vector for the observed label at time step *t*. This vector reflects the probabilities of each label being observed from a fixed label set $Z^{\prime }$, which consists of the true output sequence *Z* and a blank character ε. All paths of concatenated observed labels across time are defined as *f*.

The probability calculation process for the feasible path *f* is illustrated in Equation (12):

(12) $$P\left(f|X\right)=\overset{T}{\underset{t=1}{\Pi }}c_{f_{t}}^{t},\forall f\in {Z\prime }^{T}$$

The central principle of the CTC algorithm is to learn the direct mapping between the input and output sequences through a dynamic programming approach. To formalize this mapping, a many-to-one function β is defined, which captures all correspondences from the feasible path *f* to the true sequence *Z*. This function enhances clarity by eliminating duplicate labels and blank frames, thereby streamlining the relationship. The conditional probability of the target sequence, defined as the sum of the probabilities of all feasible paths that can generate the given target sequence P(Z|X), is expressed in Equation (13):

(13) $$P\left(Z|X\right)=\sum_{f\in \beta^{-1}\left(Z\right)}P(f|X)$$

Furthermore, the CTC loss function can be expressed as follows in Equation (15):

(14) $$L_{ctc}=-logP(f|X)$$

## 3. MPSA-Conformer-CTC/Attention

By comparing CTC-based models and Attention-based models, it can be concluded that CTC models have advantages in terms of model latency and training difficulty. However, the assumption of conditional independence in CTC models leads to poor recognition rates when an additional language model is not used, and incorporating such a model increases the workload. The RNN-T model is an improvement over the CTC model, where the prediction network can serve as a language model; however, it employs recurrent neural networks for modeling feature sequences, which can make training challenging.

In contrast, Attention-based models, from LAS to Transformer to Conformer, continuously improve recognition accuracy. Both Transformer and Conformer utilize a complete self-attention mechanism for sequence modeling and soft alignment. However, prior to decoding, they must generate all encodings before performing soft alignment and decoding, resulting in greater latency compared to CTC models that output frame-by-frame.

**Figure 3:**
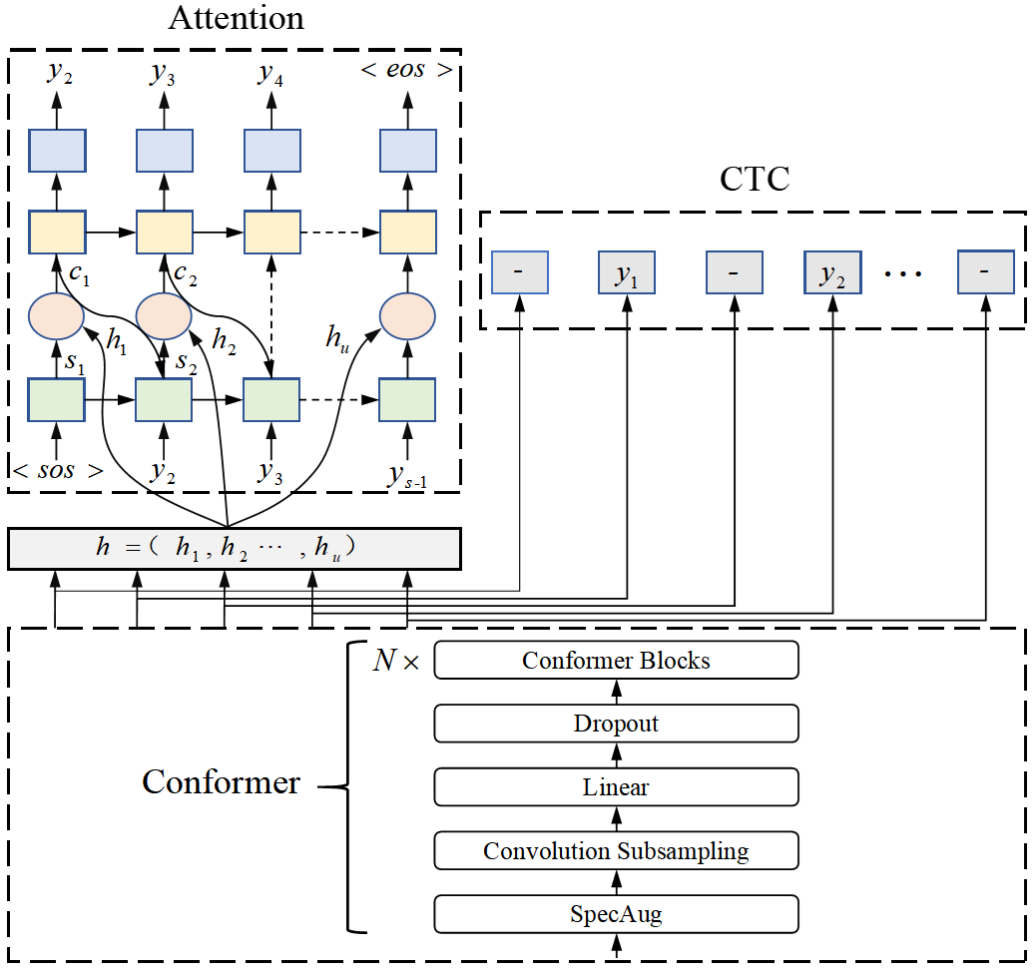
Conformer-CTC/Attention Model for Tibetan Speech Recognition.

In recent years, researchers have combined the advantages of CTC algorithms and Attention-based models, proposing a hybrid CTC/Attention joint decoding scheme [36]. Furthermore, existing studies have demonstrated that the recognition accuracy of the Conformer model surpasses that of the Transformer model [31]. Based on this analysis, the speech recognition model in this chapter will adopt the Conformer as the shared encoder in the hybrid CTC/Attention model, as illustrated in Figure 3.

The model consists of three components: a shared encoder, a CTC decoder, and an attention decoder. The shared encoder is composed of *N* Conformer encoder layers. Initially, the model processes 80-dimensional Fbank features and 3-dimensional fundamental frequency features as input, which undergo data augmentation. The augmented acoustic features are then downsampled using convolutional layers to reduce dimensionality and capture local information effectively. Subsequently, the features are transformed through a linear mapping layer, and regularization techniques are applied to prevent overfitting. The processed features are fed into the encoder, which comprises multiple Conformer blocks. The CTC decoder can automatically align input and output sequences during training, although it operates under the constraint of the independence assumption. In contrast, the attention decoder learns the dependencies between target sequences without imposing any independence assumptions among characters. We employ the CTC decoder as an auxiliary training task, jointly training and optimizing it alongside the attention objective function. Both decoders share a common encoder, enabling the learned features to be complementary and mutually enhancing, thereby improving the overall performance of the model.

Unlike traditional encoder–decoder models that rely solely on the attention mechanism, the CTC/Attention architecture employs the CTC loss function as an auxiliary task, training the decoder network within a multi-task learning framework [37]. This approach leverages the forward-backward algorithm of CTC to enforce monotonic alignment between the speech and label sequences, effectively addressing issues such as premature termination and repeated predictions in the attention mechanism. During training, the overall loss function $L_{MTL}$ for the joint decoder is defined as a linearly weighted combination of the CTC-based loss function $L_{ctc}$ and the attention-based loss function $L_{att}$, as expressed in Equation (15):

(15) $$L_{MTL}=\lambda L_{ctc}+\left(1+\lambda \right)L_{att}$$

λ is a tunable parameter that satisfies 0≤λ≤1; it primarily determines the weight ratio between CTC and Attention. Another advantage of the hybrid CTC/Attention model is that the Attention objective serves as an approximate word-level target, while the CTC objective functions as a sequence-level target. Consequently, this multi-objective learning framework not only aids in estimating the required alignment process but also mitigates the word-level approximation issues associated with the Attention mechanism through the use of the sequence-level CTC objective.

### 3.1. Probabilistic Sparse Self-Attention Model

The Tibetan speech recognition model used in this study employs a hybrid CTC/Attention decoding scheme with Conformer as the shared encoder. However, during the training process, the model struggles to converge when the input feature sequences are lengthy. The time and space complexity of the attention calculations in the self-attention mechanism is $O(L^{2})$, where *L* is the length of the sequence. As the sequence length increases, the computational demands become increasingly complex. Given that the acoustic feature sequences for speech are generally of considerable length, we aim to minimize computational complexity and memory usage during system operation. To address these challenges, we reference a sparse self-attention method proposed for predicting long time series [38], which assumes that the queries in the attention mechanism exhibit sparsity.

The self-attention mechanism is critical in end-to-end speech recognition, particularly in the Conformer-CTC/Attention model for Tibetan recognition. The Scaled Dot-Product Attention (SDPA) plays a key role in the implementation of Multi-Head Self-Attention (MHSA) within the Conformer framework. According to research published by Google’s machine translation team in 2017 [39], the attention mechanism is defined abstractly. It assumes that to learn the context vector from an input sequence *X* (with sequence length *L* and dimensionality *d*), a query vector *Q* is generated based on the specific task. The attention scores are computed using a scoring function that evaluates the relevance of the query vector to each input in the sequence *X* (in this study, attention scores are calculated using the scaled dot-product method). The scores are then transformed into a probability distribution between 0 and 1 using the softmax function, ensuring that the sum equals 1. Finally, the input information is weighted and summed to produce the context vector. This attention computation method is referred to as soft attention, and all attention mechanisms used in this study employ the soft attention approach [40].

(16) $$A\left(Q,K,V\right)=soft\max\left(\frac{QK^{T}}{\sqrt{d}}\right)V$$

**Figure 4:**
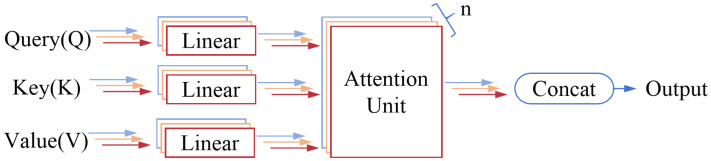
Multi-Head Attention Structure.

The attention mechanism that generates the query vector *Q* from the input sequence *X* is known as the self-attention mechanism. This mechanism typically employs a query-key-value (QKV) model, mapping the input sequence into *Q*, *K*, and *V* using different weight vectors. The attention scores computed by the scoring function reflect the relevance between *Q* and *K*. When the attention mechanism performs multiple mappings of the query-key-value vectors, it is referred to as the multi-head attention mechanism, as illustrated in Figure 4.

To further elaborate on the self-attention mechanism as described in Equation (16), let $q_{i}$, $k_{i}$, and $v_{i}$ represent the *i*-th rows of *Q*, *K*, and *V*, respectively. The attention representation of *K* with respect to $q_{i}$ can be expressed as follows:

(17) $$A\left(q_{i},K,V\right)=\sum_{j}^{L}\frac{\exp(q_{i}k_{j}^{T}/\sqrt{d})}{\sum_{l}^{L}\exp(q_{i}k_{l}^{T}/\sqrt{d})}v_{j}$$

From the calculations in Equation (17), it can be observed that the time complexity of the attention mechanism is $O(L^{2})$. This computational burden becomes significantly large as the sequence length increases, particularly when learning context vectors for long sequence speech features. Research has demonstrated that the query matrix exhibits a certain degree of sparsity [38], indicating that it is unnecessary to compute attention for all queries in the query vector. Otherwise, this results in considerable redundant computations, which have a negligible impact on the resulting attention distribution. The following analysis will first address the sparsity of the query matrix, defining the attention score distribution of *K* with respect to $q_{i}$ as expressed in Equation (18):

(18) $$p\left(k_{i}|q_{i}\right)=\frac{\exp(q_{i}k_{j}^{T}/\sqrt{d})}{\sum_{l}^{L}\exp(q_{i}k_{l}^{T}/\sqrt{d})}$$

If the distribution $p(k_{i}|q_{i})$ follows a uniform distribution *U*, then $p\left(k_{i}|q_{i}\right)=\frac{1}{L}$, which implies that the attention in Equation (18) represents the average of the value vector *V*. In this case, it becomes impossible to highlight significant contributions. Therefore, the important queries for calculating attention scores are those for which the attention score distribution $p(k_{i}|q_{i})$ deviates from uniformity. The difference between the attention score distribution and the uniform distribution can be quantified using the Kullback–Leibler divergence (i.e., relative entropy). The greater the difference between *p* and *U*, the larger the KL divergence value, as expressed in the following equation:

(19) $$KL\left(p||U\right)=\ln\sum_{j=1}^{L}e^{\frac{q_{i}k_{j}^{T}}{\sqrt{d}}}-\frac{1}{L}\sum_{j=1}^{L}\frac{q_{i}k_{j}^{T}}{\sqrt{d}}-\ln L$$

By removing the constant term from Equation (19), we obtain Equation (20), which is defined as the sparsity measure for $q_{i}$:

(20) $$M_{sparse}\left(q_{i},K\right)=\ln\sum_{j=1}^{L}e^{\frac{q_{i}k_{j}^{T}}{\sqrt{d}}}-\frac{1}{L}\sum_{j=1}^{L}\frac{q_{i}k_{j}^{T}}{\sqrt{d}}$$

The larger the sparsity measure of a query, the more important it is for calculating attention. When computing attention scores, retaining queries with a high sparsity measure while ignoring those with a low sparsity measure can accelerate the overall computation process. From a theoretical perspective, this approach is feasible; however, during training, it requires traversing all queries to compute the dot products and determine their sparsity measures before making comparisons and retaining those with higher measures. This process remains computationally intensive. To further reduce this computational burden, Equation (20) can be approximated through a sampling method, as expressed in Equation (21).

(21) $${\bar{M}}_{sparse}\left(q_{i},\tilde{K}\right)=\max_{j}\left\{\frac{q_{i}k_{j}^{T}}{\sqrt{d}}\right\}-\frac{1}{\tilde{L}}\sum_{j=1}^{L}\frac{q_{i}k_{j}^{T}}{\sqrt{d}}$$

here, $\tilde{K}$ represents the randomly sampled *K* matrix, and $\tilde{L}$ is defined by Equation (22) as the number of samples. The term $r_{sample}$ is defined as the sampling coefficient, which controls the number of samples that can be drawn.

(22) $$\tilde{L}=r_{sample}\ln L$$

After calculating the sparsity measure ${\bar{M}}_{sparse}$ for each query, only the $L_{sparse}$ queries with the highest ${\bar{M}}_{sparse}$ values are included in the attention computation. The value of $L_{sparse}$ is determined by the sparse sampling rate $r_{sparse}$, which satisfies the condition $0<r_{sparse}<1$. In this study, $r_{sparse}$ is set to 0.5 [41], as expressed in the following equation:

(23) $$L_{sparse}=r_{sparse}L$$

Therefore, the formula for the Prob-Sparse Attention method proposed in this study can ultimately be defined as Equation (24), where $I_{sparse}$ represents the indices of the $L_{sparse}$ queries. Queries not included in $I_{sparse}$ do not need to participate in the computation and can directly output their corresponding values $v_{i}$:

(24) $$\left(q_{i},K,V\right)=\left\{\begin{array}{c} \sum_{j}^{L}p\left(k_{j}|q_{i}\right)v_{j},i\in I_{sparse}\\ v_{i},else \end{array}\right.$$

### 3.2. Improvements to the CTC Algorithm Based on Maximum Entropy

This study employs a hybrid CTC/Attention decoding scheme, where the CTC component effectively addresses the alignment issues present in attention-based speech recognition methods. However, there are several challenges associated with CTC training. CTC can also be viewed as a form of multi-instance learning, where the sequence labels represent a set of all possible paths. CTC learns the probabilities of these paths through maximum likelihood estimation. As the input length increases, the number of feasible paths grows exponentially, making it difficult for CTC to identify the most suitable path.

Specifically, once CTC identifies the primary feasible path during training, erroneous information tends to cluster around this path. The predictions for this path are continuously reinforced until it completely dominates the output of the predicted results. Additionally, the introduction of blank labels in the CTC algorithm leads to an accumulation of blank labels along the path, resulting in a spike-like distribution of non-blank labels [42].

This spike distribution poses several problems, such as the potential for the model to become trapped in poor local optima during training. It also tends to generate overly confident predictions, which may not necessarily be accurate. To address these issues and inspired by the success of maximum entropy-based CTC algorithms in scene text recognition tasks [43], this study proposes an improved CTC algorithm from the perspective of maximum entropy.

Although the CTC training process continuously optimizes the loss function to guide the model’s training, the presence of numerous feasible paths may prevent the attainment of ideal high-quality alignment outputs. This phenomenon can be illustrated by the corresponding formula. Equation (25) represents the derivative of a loss function related to erroneous information concerning the variable $c_{m}^{t}$:

(25) $$\frac{\partial L_{ctc}}{\partial c_{m}^{t}}=-\frac{1}{P\left(Z|X\right)c_{m}^{t}}\sum_{\left\{f\left|f\right.\in \beta^{-1}\left(Z\right),f_{t}=m\right\}}P(f|X)$$

The above expression can be further simplified to Equation (26):

(26) $$\frac{\partial L_{ctc}}{\partial c_{m}^{t}}=-\frac{1}{P\left(Z\left|X\right.\right)c_{m}^{t}}\sum_{\left\{f\left|f\in \beta^{-1}\left(Z\right)\right.,f_{t}\in m\right\}}\gamma \left(t,k\right)\eta \left(t,k\right)$$

In the equation, $\gamma \left(t,k\right)$ represents the probability generated during the forward decoding process, while $\eta \left(t,k\right)$ denotes the backward probability. Equation (26) clearly demonstrates the proportional relationship between the scores of all feasible paths through character *m* at time step *t* and the erroneous signals. This finding indicates that once a particular feasible path dominates during training, it increases the risk of model overfitting. Although this positive feedback mechanism accelerates the convergence of training to some extent, it simultaneously restricts CTC’s ability to explore other potential feasible paths. Furthermore, even when considering various combinations of characters and time steps, this lack of diversity in the training strategy may still limit the model’s generalization capability.

Based on the above analysis, to enhance the likelihood of discovering more feasible paths and to prevent the rapid decrease of path entropy, this study presents an improved algorithm that optimizes the original CTC loss function, grounded in maximum conditional entropy. The aim is to enhance the model’s exploratory capability by incorporating the concept of conditional entropy, thereby increasing its sensitivity to different paths and potentially improving the model’s generalization performance. The improved formula is presented in Equation (27):

(27) $${L\prime }_{ctc}=L_{ctc}-\alpha Y\left(P\left(f|Z,X\right)\right)$$

In this improved algorithm, we introduce two key parameters: α and Y(P(f|Z,X)). The parameter α serves as the coefficient for maximum conditional entropy regularization, allowing us to adjust the weight of the regularization term within the loss function. In contrast, Y(P(f|Z,X)) represents the entropy of the feasible paths given the input sequence and the target sequence, effectively reflecting the degree of diversity among these paths. The specific calculation formula for these parameters is detailed in Equation (28):

(28) $$Y\left(P\left(f|Z,X\right)\right)=-\sum_{f\in \beta^{-1}\left(Z\right)}P(f|X,Z)\log P(f|X,Z)$$

Further simplification of Equation (28) yields Equation (29):

(29) $$Y\left(P\left(f|Z,X\right)\right)=-\frac{1}{P(Z|X)}\sum_{f\in \beta^{-1}\left(Z\right)}P(f|X)\log P(f|X)+\log P\left(Z|X\right)$$

Additionally, the process for computing the derivative of the entropy concerning erroneous information is illustrated in Equation (30):

(30) $$\frac{-\partial Y(P(f|Z,X))}{\partial c_{m}^{t}}=-\frac{\sum_{f\in \beta^{-1}\left(Z\right)}P(f|X)}{P\left(Z|X\right)c_{m}^{t}}(\frac{\sum_{\left\{f\left|f\in \beta^{-1}\left(Z\right),f_{t}\in m\right|\right\}}P\left(f\left|X\right.\right)\log P\left(f\left|X\right.\right)}{\sum_{f\in \beta^{-1}\left(Z\right)}P(f|X)}-\frac{\sum_{\left\{f\left|f\in \beta^{-1}\left(Z\right),f_{t}\in m\right.\right\}}P\left(f\left|X\right.\right)}{P(Z|X)})$$

From Equation (30), it is evident that the difference between P(f|X)logP(f|X) and P(f|X) scores is proportional to the erroneous signals along all feasible paths through character *m* at time step *t*. This indicates that as the score of P(f|X)logP(f|X) increases relative to that of P(f|X), the erroneous signals are correspondingly amplified. Notably, in the special case where P(f|X) equals 0, the difference between the P(f|X)logP(f|X) score and the P(f|X) score will be zero. Furthermore, when the value of P(f|X)logP(f|X) fluctuates near zero, its rate of decrease is more rapid, reaching a minimum when P(f|X)logP(f|X) equals 1.

Thus, it is clear that the probability of paths is confined within the range of 0 to $\frac{1}{e}$. During training, this results in the maximum sharing of erroneous signals among paths near the primary path, which, in turn, increases the probabilities of these nearby paths. This mechanism enhances the effectiveness of the search for feasible paths during the training process.

In this study, we subsequently integrate the aforementioned maximum conditional entropy-based CTC improvement algorithm into the Conformer-CTC/Attention model. During the training process, the final loss function of the model is determined by this algorithm, which facilitates optimization of the model’s performance. The expression for the model’s loss is presented in Equation (31):

(31) $$L_{MTL}=\lambda *\left(L_{\mathrm{ctc}}-\alpha Y\left(P\left(f|Z,X\right)\right)\right)+\left(1-\lambda \right)L_{att}$$

## 4. Experimental Results and Analysis

### 4.1. Experimental Dataset

The experimental dataset used in this study consists of two Tibetan speech recognition datasets. The first, designated as Tibetan-A, originates from the author’s laboratory. This dataset includes recordings from primary and secondary school textbooks, as well as proverbs and maxims, totaling 50,000 sentences. The audio data were collected in a dedicated recording studio, with recordings conducted by two professional broadcasters (one male and one female). The collected audio is in mono WAV format, with a sampling frequency of 16 kHz and a bit depth of 16 bits. The recorded passages were segmented based on temporal features, and a speech corpus was established by manually aligning the sentences with their corresponding audio.

The second dataset is sourced from the open-access TIBMD@MUC [44], which contains Tibetan language data. This dataset includes speech–text pairs from the three major Tibetan dialects, featuring similarities and dialect-specific variations. The text encompasses a range of content, including news articles, dialogues, novels, and poetry, amounting to a total of 23,169 sentences. The audio data were recorded by Tibetan university students from various dialect regions, totaling 50 participants. In this study, two datasets were divided into training and testing sets in a 9:1 ratio, with a portion of the training set reserved as a validation set. Detailed information is provided in Table 1.

### 4.2. Experimental Environment Configuration

The experiments in this study were conducted on the Ubuntu 20.04 operating system, utilizing the PyTorch deep learning framework. The specific configuration of the experimental environment is detailed in Table 2.

### 4.3. Configuration of Experimental Model Parameters

In this study, FBank is used as the input feature, and the Adam optimizer is employed during training, with a learning rate adaptation strategy. The Conformer encoder structure is enhanced, setting the number of encoder layers to 12, with 4 heads in the multi-head self-attention module, and the vector dimension for encoding information is set to 256. The number of units in the hidden layer is set to 2048. The Swish activation function is used, and the dropout rate is set to 0.1, with a batch size of 32. In the Transformer decoder structure, the number of decoder layers is set to 6, and the decoder output dimension, dropout rate, number of heads, and other parameters are the same as those in the encoder, also utilizing the Swish activation function.

### 4.4. Evaluation Metrics

This study employs Word Error Rate (WER) [44] as the primary evaluation metric for several key reasons. First, WER is straightforward to calculate, facilitating easy comparison and analysis across experiments. Second, it provides a comprehensive reflection of the performance of speech recognition systems, taking into account both the number of errors and the length of the output, making it a robust and effective metric. Additionally, WER intuitively highlights the real-world issues users may encounter when utilizing speech recognition systems, which is crucial for assessing the system’s practicality and user experience.

Word Error Rate (WER) is used to measure the number of errors at the word level in speech recognition models. The calculation formula is presented in Equation (32):

(32) $$WER=\frac{D+I+S}{N}\times 100\%$$

In the equation, *S* represents the number of substituted characters, *D* denotes the number of deleted characters, *I* indicates the number of additional inserted characters, and *N* refers to the total number of words in the sentence.

### 4.5. Experiments on Acoustic Feature and Modeling Unit Selection

#### 4.5.1. Acoustic Features

In speech recognition, the feature extraction step is crucial, as it involves converting the speech signal into feature vectors that can be processed by the model. The value and representational performance of the features significantly impact the classification accuracy of the model. High-quality features can greatly enhance the model’s classification accuracy, while features that are less relevant to the target task may struggle to perform effectively. Commonly used acoustic features include FBank and MFCC.

FBank is a front-end processing algorithm that simulates the nonlinear response of the human ear to sound spectra. Through steps such as pre-emphasis, framing, windowing, short-time Fourier transform, and Mel filtering, FBank can extract the spectral features of the speech signal. These features are commonly used as input for training acoustic models in speech recognition, and they can also be applied to tasks such as speech synthesis, effectively enhancing the performance of speech recognition.

MFCC (Mel-Frequency Cepstral Coefficients) is a feature defined based on the nonlinear relationship between Mel frequency and Hertz frequency, which reflects the auditory characteristics of the human ear. It extracts key information from speech by computing the Hz spectrum and is widely used in speech and speaker recognition. MFCC not only facilitates feature extraction from speech data but also effectively reduces computational dimensions; for instance, it can reduce a frame of 512-dimensional data to a more manageable 40 dimensions, thereby simplifying data processing. The calculation of this feature is performed after obtaining the FBank features of the speech signal, further deriving the MFCC features through discrete cosine transformation.

**Figure 5:**
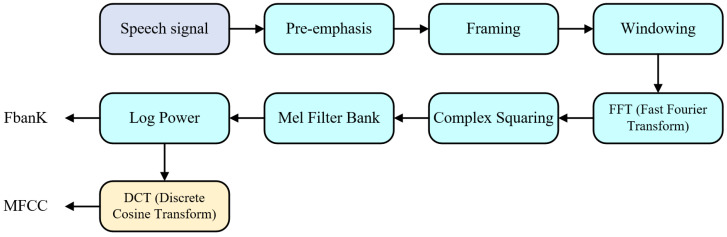
Feature Extraction Processes for FBank and MFCC.

Overall, both MFCC and FBank are acoustic features extracted based on the auditory characteristics of the human ear, playing a crucial role in speech recognition. FBank features focus more on simulating the response characteristics of the human ear, while MFCC involves further feature extraction and dimensionality reduction based on this foundation. The extraction process is illustrated in Figure 5.

#### 4.5.2. Modeling Unit

The modeling units for end-to-end Tibetan speech recognition primarily include the following components:

Syllable-Level Modeling Unit: Due to the unique bidimensional script structure of Tibetan, its writing involves both horizontal and vertical orientations. Therefore, syllables serve as an important modeling unit. In related research, the preprocessing of Tibetan text and the design of modeling methods have been completed, including the comprehensive design and extraction of syllables.

Character-Level Modeling Unit: In Tibetan, a character (Zhidin) is the fundamental unit that constitutes words, similar to how characters function in Chinese. In speech recognition models, the character-level modeling unit aids the system in more accurately recognizing and converting the various vocabularies present in the speech signal.

Component-Level Modeling Unit: Given the unique bidimensional structure of Tibetan script, which incorporates both horizontal and vertical aspects, character components are particularly crucial in the modeling process. These character components form the foundation of Tibetan writing; by recognizing these components, the model can gain a deeper understanding and analysis of Tibetan speech.

These modeling units play a key role in the end-to-end Tibetan speech recognition model, helping the model to more accurately capture and understand Tibetan speech signals, thereby achieving efficient speech recognition functionality.

This paper primarily explores the impact of FBank and MFCC features, as well as three modeling units (syllables, Character, and components) on the performance of speech recognition models. To select the final acoustic features and modeling units to be used, a comparative analysis will be conducted on the effects of FBank features and MFCC features across the three modeling units. The comparative experiments will utilize the model proposed in this paper, and the results are presented in Table 3.

According to the results shown in Table 3, in both the Tibetan-A and TIBMD@MUC datasets, the performance of the speech recognition model using FBank as the acoustic feature outperforms that using MFCC features across all three modeling units. This may be attributed to the high correlation of FBank features, which can more comprehensively preserve the spectral information of the original speech signal, thereby better reflecting the dynamic continuity of speech. Additionally, the rich information provided by FBank is more suitable for meeting the training requirements of complex deep learning models. Particularly considering the uniqueness of Tibetan, the detailed features offered by FBank enable the model to learn and recognize Tibetan speech more accurately. In contrast, MFCC features, due to their compressed nature, may fall short in expressing certain detailed information compared to FBank features.

Similarly, as evident from Table 3, selecting Zhidin as the modeling unit yields better results than using syllables, while syllables perform better than components. This is primarily because Zhidin provides fine differentiation of speech, meets the basic requirements of speech recognition, and allows for more targeted model training, making it the most effective modeling unit. Syllables, with their moderate information granularity, help simplify model complexity and strike a balance between recognition efficiency and accuracy, thus performing second best. In contrast, components yield the poorest results, likely due to their weak correspondence with pronunciation, which increases the model’s complexity when used as a modeling unit. In the subsequent experiments, the acoustic features and modeling units will both be selected as FBank and Zhidin.

### 4.6. Experiment on Optimal Joint Parameter Selection λ

To validate the effectiveness of the joint CTC/Attention approach, we first examined the performance of CTC and Attention separately on two Tibetan speech recognition datasets. We set λ=1 and λ=0 to represent the Conformer-CTC and Conformer-Attention models, respectively. To investigate the effects of different joint parameters λ, we pre-set the joint training parameter λ for the CTC/Attention model to 0.1, 0.3, 0.5, and 0.7. A comparative analysis of these four values was conducted, with the final experimental results presented in Table 4.

As shown in Table 4, the recognition performance is suboptimal when λ=0 (Conformer-Attention model) or λ=1 (Conformer-CTC model). When the joint training parameter λ exceeds 0.5, the loss weight during joint decoding primarily concentrates on the CTC decoder, which limits the ability of the Attention mechanism to leverage its contextual modeling advantages, resulting in unsatisfactory decoding performance. Conversely, when λ=0.3, the loss during the joint decoding process mainly focuses on the Attention decoder. In this case, CTC serves as an auxiliary task, effectively accelerating the alignment and decoding processes, leading to the best decoding performance. This outcome demonstrates the effectiveness of the proposed method.

**Figure 6:**
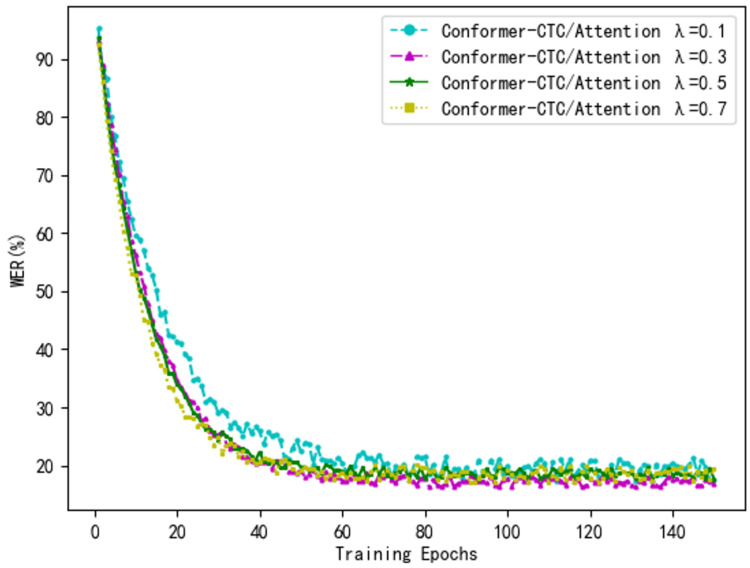
Model Error Rate Variation Curve.

Figure 6 illustrates the variation in model error rates on the Tibetan-A dataset for different values of λ. The model achieves optimal performance when λ=0.3, and as the value of λ increases, the convergence speed of the model noticeably accelerates. This acceleration is attributed to the CTC model’s backpropagation algorithm, which can directly update the model parameters. However, when λ is set to 0.5 or 0.7, although the convergence speed is rapid, the accuracy declines. In these cases, the high proportion of CTC in the multi-task learning setup adversely affects the convergence properties due to the conditional independence assumptions of the model, potentially leading to issues such as overfitting.

From the analysis of Table 3 and Figure 6, it can be observed that when the joint training parameter λ is set to 0.3, the hybrid CTC/Attention model demonstrates optimal performance, achieving the highest recognition rate and a faster convergence speed.

### 4.7. Comparative Experiments of Different Optimization Strategies

To validate the effectiveness of the optimization techniques employed in this study, we used the proposed Conformer-CTC/Attention model as a baseline for comparative experiments against algorithm-optimized models on two Tibetan speech datasets. The model labeled as +Prob-Sparse employs a probability-sparse self-attention mechanism, while +Max-Entro indicates improvements based on a maximum entropy CTC algorithm. MPSA-Conformer-CTC/Attention refers to the Conformer-CTC/Attention model enhanced with both the probability-sparse self-attention and the maximum entropy algorithm. Table 5 presents a comparison between the baseline model and the algorithm-optimized models across these two speech datasets.

The four models were tested on the Tibetan-A and TIBMD@MUC2 datasets. As shown in Table 5, the WER of Model 2, Conformer-CTC/Attention (+Prob-Sparse), significantly decreased compared to the baseline model, with rates of 0.5% and 6%, respectively. Additionally, the computational complexity of the model reduced from O($N^{2}$) to O(*N*), demonstrating the effectiveness of the proposed probability-sparse self-attention mechanism. Model 3, Conformer-CTC/Attention (+Max-Entro), exhibited a modest decrease in WER relative to the baseline, with rates of 3.3% and 9.1%, indicating that the improvements based on the maximum entropy CTC algorithm are effective. Overall, there is an enhancement in the speech recognition rate of the Conformer-CTC/Attention model. Model 4, MPSA-Conformer-CTC/Attention, showed a significant decrease in WER compared to the baseline model, with rates of 10.68% and 9.57%, while the model’s complexity also decreased to O(*N*). Experimental results confirm that the improved model not only reduces memory consumption and training time but also enhances the model’s generalization ability and accuracy.

**Figure 7:**
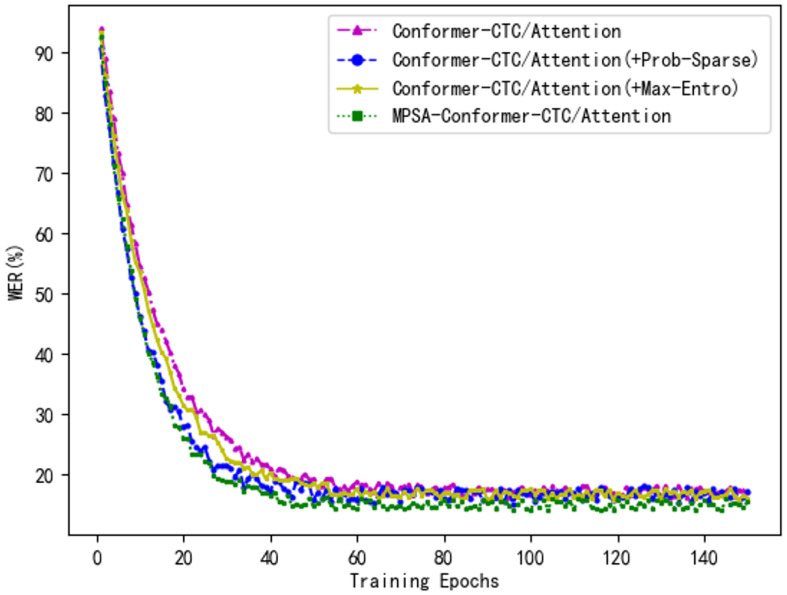
Comparison of the Variation Curves of Error Rates During Optimized Model Training.

Figure 7 illustrates the error rate curves of different optimized models during the training process on the Tibetan-A dataset. It is evident from the figure that by the 15th epoch, Model 2, Conformer-CTC/Attention (+Prob-Sparse), demonstrates a clear advantage over the baseline model, exhibiting a steeper slope and a faster decline in the error rate. This indicates that Model 2 can achieve a lower loss value more quickly and stably compared to the baseline model. Although Model 3, Conformer-CTC/Attention (+Max-Entro), ultimately achieves a lower error rate, its training speed shows no significant improvement. Model 4, MPSA-Conformer-CTC/Attention, not only accelerates model convergence but also enhances model accuracy. Experimental results confirm that our approach improves both the accuracy and efficiency of the model.

### 4.8. Comparative Experiments with Advanced Tibetan Speech Recognition Models

This study compares our model, MPSA-Conformer-CTC/Attention, with four mainstream Tibetan speech recognition models: the BLSTM-CTC model [19], DFCNN-CTC model [22], Wavenet-CTC model [24], and MRDCNN-CTC model [29], using the Tibetan-A dataset. The specific experimental results are presented in Table 6.

In this study, we conducted extensive comparisons and analyses of Tibetan speech recognition models. By evaluating the performance of different models on the Tibetan-A and TIBMD@MUC datasets, we identified several key findings. Specifically, the BLSTM-CTC model exhibited a Word Error Rate (WER) of 21.58% on the Tibetan-A dataset, while it achieved a WER of 35.59% on the more challenging TIBMD@MUC dataset. The DFCNN-CTC model showed slightly better performance on the Tibetan-A dataset, with a WER of 20.42%, but it recorded a WER of 28.69% on the TIBMD@MUC dataset. The Wavenet-CTC model performed somewhat worse than the other models, achieving a WER of 22.33% on the Tibetan-A dataset and 32.48% on the TIBMD@MUC dataset. In contrast, the MRDCNN-CTC model demonstrated superior performance on the Tibetan-A dataset, with a WER of 17.57% and a WER of 27.89% on the TIBMD@MUC dataset. Notably, our proposed OURs model achieved the lowest WERs on both the Tibetan-A and TIBMD@MUC datasets, at 15.38% and 22.94%, respectively, indicating outstanding performance. Overall, this study provides valuable insights for enhancing Tibetan speech recognition performance, and future work could further explore and optimize the OURs model to improve its performance and generalization capabilities.

## 5. Discussion and Results

In this study, our primary objective was to enhance the performance of Tibetan speech recognition systems, specifically addressing the challenges of accuracy and computational complexity highlighted in the introduction. We successfully developed an innovative end-to-end network architecture, the MPSA-Conformer-CTC/Attention model, which integrates the strengths of the Conformer encoder and the joint CTC/Attention decoder. This model significantly improved the extraction of global features from speech and optimized the decoding process.

To tackle convergence issues during training, we implemented the Prob-Sparse Attention method. This approach not only reduced computational complexity and memory usage but also enhanced the model’s performance and stability, enabling it to manage long sequence inputs more effectively. Furthermore, we introduced a maximum entropy-based CTC improvement algorithm, which addressed critical challenges associated with the traditional CTC algorithm, including the increase in path count, spike distribution, and local optima. This optimization led to greater robustness and training efficiency of the model.

The results obtained indicate that our proposed methodologies yield significant advancements in Tibetan speech recognition technology. These findings not only contribute to the specific challenges faced in this domain but also provide valuable insights that can be applied to other areas of speech recognition research.

Looking ahead, future research steps will focus on further refining the model architecture and exploring additional techniques to enhance performance, such as incorporating more extensive datasets and experimenting with different training paradigms. Additionally, we aim to investigate the applicability of our methodologies to other languages and dialects, thus broadening the impact of our research in the field of speech recognition.

## Figures and Tables

**Table 1:** Detailed Information on the Experimental Dataset.

Dataset	Data Partitioning	Number of Corpus Instances	Duration of Sentences (h)
Tibetan-A	Training Set	39,060	27.53
	validation set	5940	13.84
	Testing Set	5000	12.2
TIBMD@MUC	Training Set	16,749	14.76
	validation set	3845	8.24
	Testing Set	2575	7

**Table 2:** Experimental Environment Configuration.

Name	Model/Parameter
Linux Version	5.11.0
Operating System	Ubuntu 20.04
GPU	8 × RTX3090
CUDA Version	11.8
Deep Learning Framework	PyTorch 2.1
Programming Language	Python 3.8

**Table 3:** Comparative Analysis of the Impact of Acoustic Features and Modeling Units on Model Performance.

Dataset	Acoustic Features	Modeling Unit	WER (%)
Tibetan-A	MFCC	Components	22.37
		Syllables	20.25
		characters	19.42
	Fbank	Components	19.28
		Syllables	18.33
		characters	**17.22**
TIBMD@MUC	MFCC	Components	29.21
		Syllables	27.89
		characters	27.33
	Fbank	Components	27.74
		Syllables	26.30
		characters	**25.32**

**Table 4:** Word Error Rate (WER) for Different Models.

Index	Model	WER (Tibetan-A) %	WER (TIBMD@MUC) %
1	Conformer-CTC, λ=1	19.68	28.35
2	Conformer-Attention, λ=0	21.92	28.28
3	Conformer-CTC/Attention, λ=0.1	19.33	27.19
4	Conformer-CTC/Attention, λ=0.3	**17.22**	**25.37**
5	Conformer-CTC/Attention, λ=0.5	18.64	26.47
6	Conformer-CTC/Attention, λ=0.7	19.17	27.39

**Table 5:** Comparison of WER for Different Models.

Index	Model	WER (Tibetan-A) %	WER (TIBMD@MUC) %	Complexity
1	Conformer-CTC/Attention	17.22	25.37	O($N^{2}$)
2	Conformer-CTC/Attention (+Prob-Sparse)	17.13	23.84	O(*N*)
3	Conformer-CTC/Attention (+Max-Entro)	16.65	23.05	O($N^{2}$)
4	MPSA-Conformer-CTC/Attention	15.38	22.94	O(*N*)

**Table 6:** Results of Comparative Experiments with Advanced Tibetan Speech Recognition Models.

Index	Model	WER (Tibetan-A) %	WER (TIBMD@MUC) %
1	BLSTM-CTC [19]	21.58	35.59
2	DFCNN-CTC [22]	20.42	28.69
3	Wavenet-CTC [24]	22.33	32.48
4	MRDCNN-CTC [29]	17.57	27.89
5	MPSA-Conformer-CTC/Attention	**15.38**	**22.94**

## Data Availability

The data provided in this study can be obtained by contacting the corresponding author. Due to ongoing experiments in the laboratory, the data cannot be made publicly available at this time.

## Abbreviations

The following abbreviations are used in this manuscript:

CTC	Connectionist Temporal Classification
MPSA	MaxEnt-Optimized Probabilistic Sparse Attention
ASR	Automatic Speech Recognition
E2E	End-to-End
AED	Attention-based Encoder–Decoder
RNN	Recurrent Neural Networks
BLSTM	Bidirectional Long Short-Term Memory
WER	Word Error Rate
CNN	Convolutional Neural Network
CER	Character Error Rate
HMM	Hidden Markov Model
MRDCNN	Multi-Resolution Dual Convolutional Neural Network
LAS	Attend and Spell
SDPA	Scaled Dot-Product Attention
MHSA	Multi-Head Self-Attention
FBank	Filter Bank
MFCC	Mel-Frequency Cepstral Coefficients
Prob-Sparse	Probability-sparse self-attention
Max-Entro	Maximum Entropy
DFCNN	Deep Filtered Convolutional Neural Network
